# Investigation of a Cluster of Sequence Type 22 Methicillin-Resistant *Staphylococcus aureus* Transmission in a Community Setting

**DOI:** 10.1093/cid/cix539

**Published:** 2017-10-25

**Authors:** Michelle S Toleman, Emmeline R Watkins, Tom Williams, Beth Blane, Belinda Sadler, Ewan M Harrison, Francesc Coll, Julian Parkhill, Bernadette Nazareth, Nicholas M Brown, Sharon J Peacock

**Affiliations:** 1 University of Cambridge; 2 Wellcome Trust Sanger Institute, Hinxton; 3 Cambridge University Hospitals NHS Foundation Trust; 4 Health Protection Team, Public Health England–East of England, Thetford; 5 Infection Prevention and Control, Cambridgeshire and Peterborough Clinical Commissioning Group; 6 London School of Hygiene and Tropical Medicine; 7 Clinical Microbiology and Public Health Laboratory, Public Health England, Cambridge, United Kingdom

**Keywords:** MRSA, epidemiology, community, general practice, genome sequencing

## Abstract

**Background:**

Whole-genome sequencing (WGS) has typically been used to confirm or refute hospital/ward outbreaks of methicillin-resistant *Staphylococcus aureus* (MRSA) identified through routine practice. However, appropriately targeted WGS strategies that identify routinely “undetectable” transmission remain the ultimate aim.

**Methods:**

WGS of MRSA isolates sent to a regional microbiological laboratory was performed as part of a 12-month prospective observational study. Phylogenetic analyses identified a genetically related cluster of E-MRSA15 isolated from patients registered to the same general practice (GP) surgery. This led to an investigation to identify epidemiological links, find additional cases, and determine potential for ongoing transmission.

**Results:**

We identified 15 MRSA-positive individuals with 27 highly related MRSA isolates who were linked to the GP surgery, 2 of whom died with MRSA bacteremia. Of the 13 cases that were further investigated, 11 had attended a leg ulcer/podiatry clinic. Cases lacked epidemiological links to hospitals, suggesting that transmission occurred elsewhere. Environmental and staff screening at the GP surgery did not identify an ongoing source of infection.

**Conclusions:**

Surveillance in the United Kingdom shows that the proportion of MRSA bacteremias apportioned to hospitals is decreasing, suggesting the need for greater focus on the detection of MRSA outbreaks and transmission in the community. This case study confirms that the typically nosocomial lineage (E-MRSA15) can transmit within community settings. Our study exemplifies the continued importance of WGS in detecting outbreaks, including those which may be missed by routine practice, and suggests that universal WGS of bacteremia isolates may help detect outbreaks in low-surveillance settings.

## Teaser

This study identifies a community cluster of methicillin-resistant *Staphylococcus aureus* transmission missed by routine practice and provides genomic evidence for transmission of a typically nosocomial lineage within community rather than hospital settings. Systematic whole-genome sequencing may increase detection of such outbreaks.

In the United Kingdom, the emergence of the epidemic methicillin-resistant *Staphylococcus aureus* (EMRSA) 15 lineage (multilocus sequence type [ST] 22) in 1991 was followed by its rapid dissemination throughout UK hospitals and long-term care facilities (LTCFs) [1–4]. This was associated with a dramatic increase in the rate of MRSA bacteremia until rates began to decline in the mid-2000s [5]. Voluntary reporting of MRSA bacteremia in England was replaced by mandatory reporting in 2001 [6], to which mandatory enhanced epidemiological surveillance was added in 2005 [7]. Since 2013, investigation of MRSA bacteremia requires a locally administered postinfection review (PIR), which aims to identify how the case occurred and preventive actions to avoid recurrence. Consequently, responsibility for cases are attributed to the organization best placed to implement these actions [8].

Currently, only 40% of these reported bacteremia cases are attributed to a hospital, which suggests that transmission outside of hospitals is a substantial contributor to overall MRSA bacteremia rates [9]. Definitive evidence for community transmission as a driver of MRSA infection in the United Kingdom is limited, but is supported by a recent epidemiological and bacterial genomic survey that captured transmission events over a 16000-km^2^ area of the East of England [10]. This argued against conventional wisdom that ST22 is largely healthcare associated in the United Kingdom [1, 11], and provided evidence for a substantial burden of MRSA transmission outside of hospital settings (ie, in the community).

Despite this, the focus on MRSA prevention and control remains hospital-centric. Here, we characterize the community-based transmission of EMRSA-15 (typically considered a nosocomial lineage) in a general practice (GP) surgery. This study argues for a renewed focus on infection control in community settings and demonstrates the role of bacterial whole-genome sequencing (WGS) in community MRSA surveillance and infection control.

## METHODS

### Study Design

A cluster of 13 MRSA-infected individuals registered to a single GP surgery in Cambridgeshire was first detected during a 12-month prospective study of all MRSA-positive samples processed by the Public Health England (PHE) Clinical Microbiology and Public Health Laboratory, Cambridge University Hospitals NHS Foundation Trust (CUH) in Cambridge, United Kingdom. This study has been described in detail elsewhere [10]. In brief, 1465 individuals were identified with MRSA isolated at least once from either screening swabs and/or clinical specimens, and WGS of 2282 MRSA isolates from these cases. Combined analysis of WGS data revealed a single large cluster of closely related MRSA (defined based on a pairwise single-nucleotide polymorphism [SNP] distance <50 SNPs) that contained 22 isolates from these 13 individuals. This formed the starting point for a public health investigation and the study described here.

### Public Health Investigation

The detection of the MRSA cluster resulted in an investigation conducted in May 2015 by the local PHE health protection team. The GP surgery had >10000 registered patients and provided specialist services including diabetic and podiatry clinics. The 13 people involved in the MRSA cluster (defined as cases) were sent an information sheet and details of opt-out consent prior to individual GP record review. If consent was not withheld and records were available, data were collected on demographics, comorbidities, and date of first MRSA detection. In the 6 months prior to each patient’s first recorded positive MRSA result, healthcare attendance (primary care, hospital outpatient, or inpatient) and microbiological samples that were MRSA negative were recorded. Incidence rates of MRSA-positive individuals were calculated per 10000 registered patients at the study surgery. The CUH laboratory information system was used to determine incidence of MRSA positivity based on samples submitted to CUH from 4 comparable practices within the same region (defined as practices with >10000 registered patients in the same GP classification group) [12]. All data were collected and analyzed within the context of the public health investigation.

Staff at the GP surgery were invited to undergo MRSA screening (nose/throat/groin swabs) following attendance at an information session and written consent. Environmental MRSA screening was performed at 40 sampling points in the building. Samples were taken from high-contact equipment and surfaces in the following areas: 2 randomly selected medical clinic consultation rooms, 2 nursing clinic rooms (where the ulcer clinic, which was the strongest epidemiological link between patients, was held), and shared patient waiting areas. At each sampling point, an area of approximately 10 cm × 10 cm (or entire surface of handles) was swabbed and cultured for MRSA using direct plating onto chromogenic agar [13].

Extended case finding was performed to identify further cases that might be linked to the cluster over a longer time period, and for whom MRSA isolates had been stored and could be retrieved for sequencing. This involved 3 different approaches (1) A retrospective search was performed of the CUH information system for MRSA-positive samples submitted by the GP surgery between January 2006 and June 2015. These data were then cross-referenced with the bacterial archive database to determine if isolates had been stored at –80°C. (2) Laboratory surveillance was conducted in the laboratory between November 2015 and February 2016 to detect MRSA-positive individuals from the GP surgery. (3) Recent PIRs at the GP surgery were reviewed. Isolates were requested from the receiving hospital for WGS and patient records reviewed as described above.

### Whole-Genome Sequencing, Typing, and Data Analysis

DNA was extracted, libraries were prepared, and 150-bp paired end sequences were determined on an Illumina HiSeq2000 (original study isolates) or MiSeq (isolates identified through additional case finding). Methods were as previously described [14]. Details of reads and depth of coverage/N50 are provided in Supplementary Table 1. Sequence data were submitted to the European Nucleotide Archive (www.ebi.ac.uk/ena; accession numbers are also listed in Supplementary Table 1). STs were assigned using sequence data, an in-house script, and the multilocus sequence type (MLST) database (http://saureus.mlst.net/), and STs were assigned to clonal complexes (CCs). Isolates were mapped using SMALT (http://www.sanger.ac.uk/science/tools/smalt-0) to the E-MRSA15 reference genome (strain HO 5096 0412, accession number HE681097). Mobile genetic elements, indels, and regions of high-density SNPs were excluded to identify the phylogenetically informative core genome for each isolate, and SNPs were used to create a midpoint-rooted, maximum-likelihood phylogeny using RAxML with 100 bootstraps [15]. Trees were visualized using Figtree (http://tree.bio.ed.ac.uk/software/figtree/) and ITOL (http://itol.embl.de/). In silico polymerase chain reaction of the variable X-region of the *spa* gene was undertaken using the genome data and published primers [16]. Spa type was then determined using SpaTyper (http://spatyper.fortinbras.us).

### Ethical Considerations

Study protocol approval for the prospective study was granted by the National Research Ethics Service (reference (11)/EE/0499), the National Information Governance Board Ethics and Confidentiality Committee (reference ECC 8-05(h)/2011), and the Cambridge University Hospitals NHS Foundation Trust Research and Development Department (reference A092428). The 13 people involved in the MRSA cluster were sent an information sheet and details of opt-out consent prior to individual GP record review. All data were collected and analyzed within the context of the public health investigation.

## RESULTS

**Figure 1.**
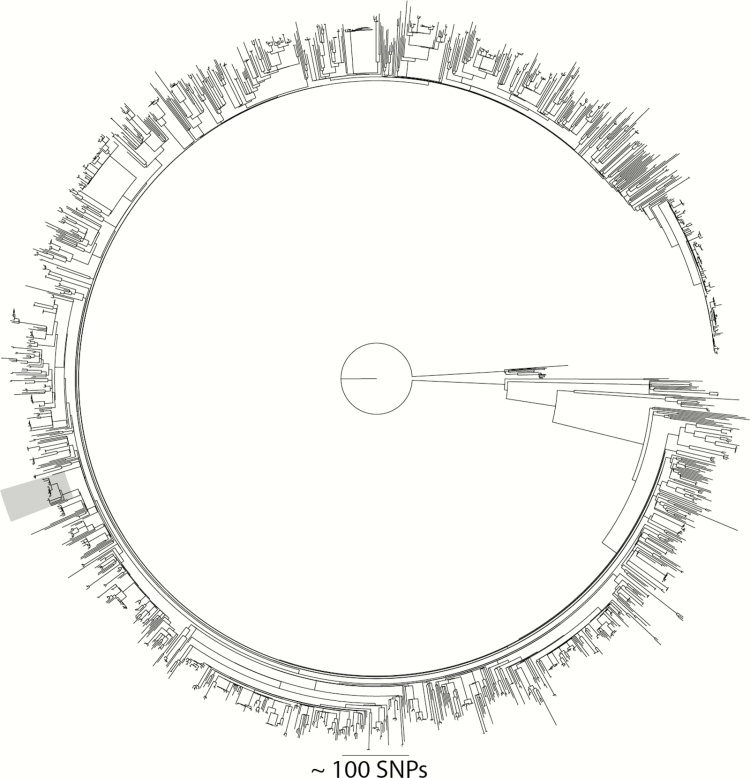
Maximum likelihood tree generated from single-nucleotide polymorphism (SNP) sites in the core genome for 1715 CC22 isolates from the 2012–2013 study. The clade highlighted in gray is the largest cluster (with a maximum SNP cutoff of 50) within the collection, and represents patients registered to the study general practice surgery.

**Figure 2.**
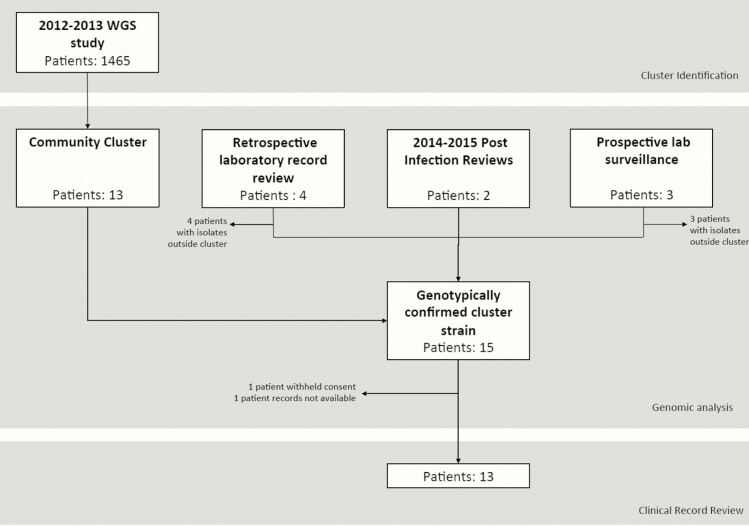
Flow diagram summarizing patient identification. One patient was captured by both the community cluster and extended retrospective laboratory record review. Abbreviation: WGS, whole-genome sequencing.

During the year-long prospective MRSA study of carriage and clinical MRSA isolates in the East of England between April 2012 and April 2013, we identified a number of potential outbreaks based on genomic relatedness and epidemiological links [10]. One potential outbreak consisted of 13 MRSA-positive individuals (22 isolates) registered with the same GP surgery in Cambridgeshire and therefore was of particular interest. All 13 isolates were ST22 and part of the EMRSA-15 clade (Figure 1). We initiated an investigation to rule out ongoing transmission, and to elucidate if this represented community-based transmission or “spill-over” from a hospital/LTCF. Extended case finding identified additional MRSA-positive individuals attending the same GP with samples available for sequencing (Figure 2). First, retrospective review of electronic laboratory records identified 4 individuals with a total of 7 isolates retrievable for sequencing, one of whom (patient [P] 04) had already been identified in the initial 13 cases. Second, prospective surveillance of MRSA-positive samples sent from the GP surgery over 3 months between November 2015–February 2016 and surveillance of new positive MRSA samples by the infection control team identified 3 retrievable isolates from 3 individuals. Third, 2 PIRs had been undertaken in 2014–2015 (P12/P13). Both patients had died with MRSA bacteremia in another regional hospital. A single isolate from each blood culture was obtained for each patient from the admitting hospital. A summary of the 22 patients (34 isolates) from the original study and additional case finding is provided in Table 1. The median number of MRSA isolates per patient was 1 (range, 1–4). Four patients had only screening samples submitted. Of those clinical samples submitted, 61% were reported as superficial swabs of lower limbs/foot, while 3 were from blood cultures and 1 from pus (all from different patients).

**Table 1.** Patient and Sample Information

Study ID	Isolation Year	Sample Type/Site	Method of Identification	MLST	Within Phylogenetic Cluster?	Included in Public Health Investigation?
P01_1	2012	Clinical, foot	Coll et al, 2017	22	Yes	Yes
P02_1	2012	Clinical, leg	Coll et al, 2017	22	Yes	Yes
P02_2	2013	Clinical, leg	Coll et al, 2017	22	Yes	Yes
P03_1	2012	Clinical, foot	Coll et al, 2017	22	Yes	Yes
P03_2	2012	Screen	Coll et al, 2017	22	Yes	Yes
P04_1	2012	Clinical, leg	Coll et al, 2017	22	Yes	Yes
P04_2	2015	Clinical, ankle	This study, laboratory record review	22	Yes	Yes
P04_3	2014	Screen	This study, laboratory record review	22	Yes	Yes
P04_4	2014	Screen	This study, laboratory record review	22	Yes	Yes
P05_1	2012	Clinical, unspecified	Coll et al, 2017	22	Yes	Yes
P06_1	2012	Clinical, leg	Coll et al, 2017	22	Yes	Yes
P06_2	2012	Screen	Coll et al, 2017	22	Yes	Yes
P07_1	2012	Clinical, leg	Coll et al, 2017	22	Yes	Yes
P07_2	2012	Clinical, leg	Coll et al, 2017	22	Yes	Yes
P07_3	2012	Clinical, leg	Coll et al, 2017	22	Yes	Yes
P08_1	2012	Clinical, foot	Coll et al, 2017	22	Yes	Yes
P09_1	2012	Clinical, leg	Coll et al, 2017	22	Yes	Yes
P10_1	2012	Screen	Coll et al, 2017	22	Yes	Yes
P11_1	2013	Clinical, leg	Coll et al, 2017	22	Yes	Yes
P11_2	2013	Screen	Coll et al, 2017	22	Yes	Yes
P12_1	2014	Clinical, blood	This study, PIR	22	Yes	Yes
P13_1	2015	Clinical, blood	This study, PIR	22	Yes	Yes
P14_1	2012	Clinical, leg	Coll et al, 2017	22	Yes	No
P14_2	2012	Clinical, unspecified	Coll et al, 2017	22	Yes	No
P15_1	2012	Clinical, back	Coll et al, 2017	22	Yes	No
P15_2	2012	Screen	Coll et al, 2017	22	Yes	No
P15_3	2013	Screen	Coll et al, 2017	22	Yes	No
P16_1	2014	Clinical, genital	This study, laboratory record review	6	No	No
P17_1	2014	Clinical, finger	This study, laboratory record review	45	No	No
P18_1	2014	Screen	This study, laboratory record review	22	No	No
P19_1	2015	Screen	This study, laboratory record review	45	No	No
P20_1	2015	Screen	This study, prospective surveillance	45	No	No
P21_1	2016	Clinical, blood	This study, prospective surveillance	1539	No	No
P22_1	2016	Clinical, abscess	This study, prospective surveillance	22	No	No

Abbreviations: MLST, multilocus sequence type; P, patient; PIR, postinfection review.

### Genetic Analysis

**Figure 3.**
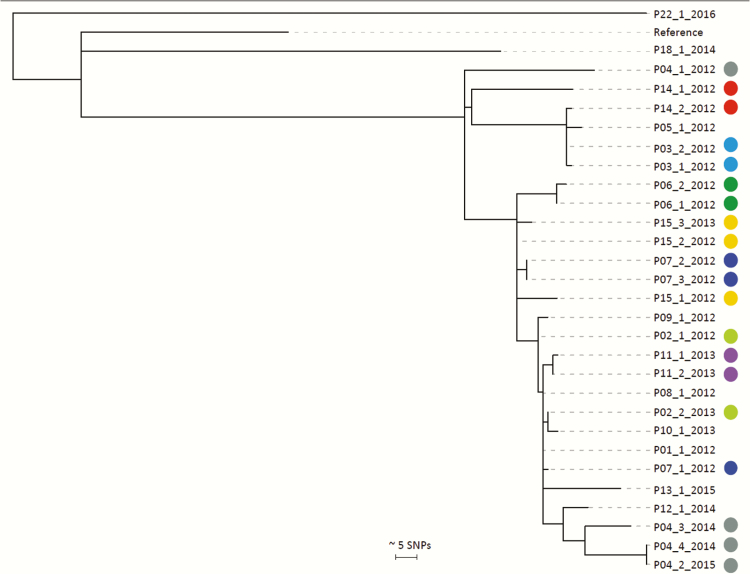
Phylogenetic analyses of 29 methicillin-resistant *Staphylococcus aureus* sequence type 22 isolates from 15 cases linked to a general practice surgery. Midpoint rooted maximum likelihood tree based on single-nucleotide polymorphisms in the core genome. Each isolate is labeled as patient (P) study number_isolate number_year of isolation. Circles indicate multiple isolates from the same patient, with each color being unique to a patient. Cases without circles signify from patients those with a single isolate only. Abbreviation: SNP, single-nucleotide polymorphism.

Spa genotyping showed that the cluster was formed of 2 main *spa* types (t032, t294) with 3 additional variants (Supplementary Figure 1). STs were derived from WGS data for the 12 MRSA isolates identified through additional case finding. The predominant ST was ST22 (7 isolates, 3 individuals), the remainder being ST45 (3 isolates, 3 individuals), ST6 (1 isolate), and ST1539 (a single-locus variant of ST221, 1 isolate). The non-ST22 cases were excluded from further analysis. After combining the 22 ST22 isolates from the original study and 7 from additional case finding to a total of 29 ST22 isolates, a maximum-likelihood tree was constructed based on SNPs in the core genome compared to the EMRSA-15 reference genome. This demonstrated clustering of 27 of the 29 ST22 isolates from 15 individuals (Figure 3), now referred to as cases. Of these, 13 had been identified in the original cluster and the additional 2 isolates were from 2 cases (P12 and P13) identified during PIRs of bloodstream infections. The median pairwise SNP distance between the 27 cluster isolates from these 15 cases was 21 (range, 0–58; interquartile range [IQR], 10–37). The median pairwise SNP distance for cluster isolates from the same person (in 8 cases with >1 isolate) was 5 (range, 0–60; IQR, 1.5–15.5). One patient (P04) had cluster isolates that extended over a period of 34 months (a basal isolate in 2012, and 3 isolates in 2014–2015 with pairwise distances of 60, 59, and 57 SNPs from the 2012 isolate).

### Public Health Investigation

To further understand the cluster, a public health investigation was performed to investigate the 15 genomically linked cases, together with screening of staff and the environment for the presence of MRSA. Two of the 15 cases (P14/P15) were excluded from further epidemiological analysis due to missing patient records or refusal of consent. Of the remaining 13 cases, the median age was 80 years (range, 12–91; IQR, 61.5–81.5) at the time of the investigation, and 6 cases (46%) were women. Geographical mapping of first MRSA isolation date and place of residence for each case demonstrated that cases lived within 5.6 km of each other and 2 individuals (P10/P11) lived on the same street. No cases lived in the same household or LTCF.

**Figure 4.**
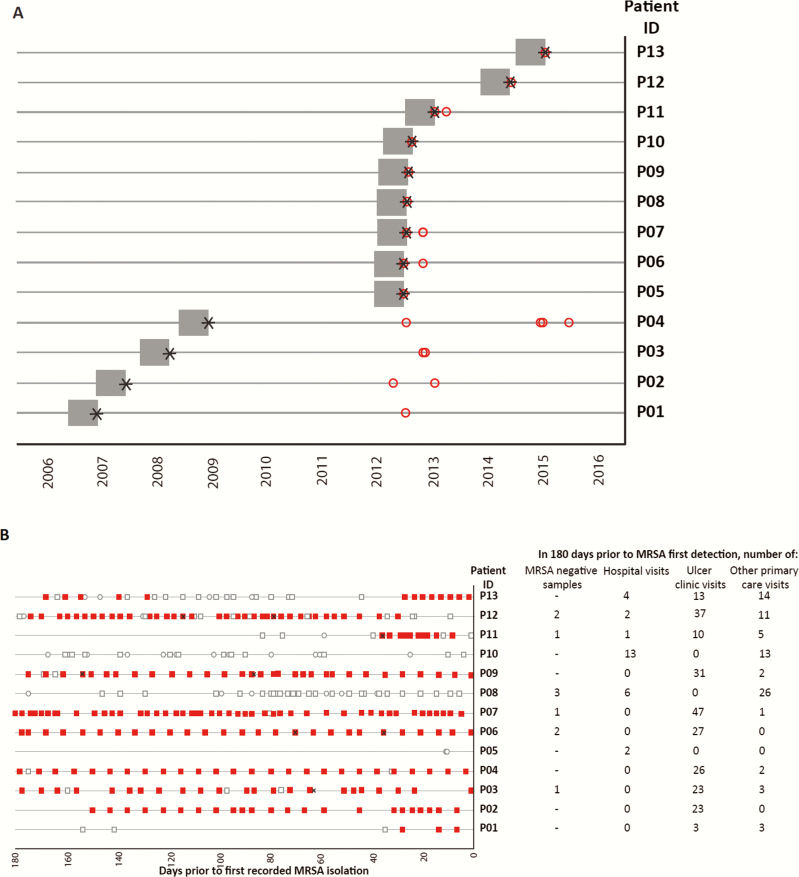
*A*, Date of first known positive methicillin-resistant *Staphylococcus aureus* (MRSA) sample (denoted by black star) for 13 individuals investigated in public health investigation, and the preceding 6-month window (gray boxes) during which contacts with healthcare for each case were established. Red open circles denote date of genomically confirmed cluster lineage MRSA samples for each individual. *B*, Timeline summarizing healthcare contact for 13 cases in the 6 months prior to first MRSA-positive sample. The timeline for each case does not necessarily overlap, and ranges between 2006 and 2015. Recorded contact with healthcare represented as follows: open circle, hospital; red square, ulcer clinic; open square, any other general practice visit. Black crosses indicate date of negative MRSA sample.

Review of sample requesting information showed a predominance of lower limb swabs (cases with samples including lower limb, 9; screen alone, 1; bacteremia alone, 2). GP medical records revealed that the date of first recorded MRSA-positive sample for cases ranged from 2006 to 2015 (Figure 4A). Healthcare contact by each case in the 6 months prior to first MRSA detection was extensive for all but 2 patients (Figure 4B). Six of the 13 cases had attended hospital in this period, of whom 3 cases (P08/P11/P12) had attended only 1 hospital, 2 cases (P05/P13) had attended 2 different hospitals, and 1 case (P10) had attended 3 different hospitals. Crucially, no overall link could be made between cases and attendance at a hospital (Figure 4A). Six individuals had 1 or more samples that were negative for MRSA in the 6 months prior to their MRSA first detection date, and had no record of hospital attendance in the intervening time. Eleven of the 13 cases had attended the GP leg ulcer clinic. P5 and P11 had not, but P11 lived on the same road as P10.

A total of 57 GP surgery staff (approximately 90% of current clinical/nonclinical employees) received multisite MRSA screens, all of which were negative. This included 4 nurses who had worked at the ulcer clinic since the first positive MRSA samples in 2008. Forty environmental samples were taken from communal waiting areas and clinic rooms, all of which were also MRSA negative.

Given that this cluster was only identified fortuitously by genome sequencing, we sought to determine if the incidence rate in the practice had been higher than that expected. This was achieved by comparing the incidence rate of MRSA-positive samples submitted to the CUH diagnostic microbiology laboratory between 2006 and 2013 between the study GP surgery and 4 other practices of a similar size and patient demographic within Cambridgeshire. This showed a fluctuating rate over time for all 4 practices, with no identifiable outbreak signal for the general practice under investigation (Supplementary Figure 2).

## DISCUSSION

In this study, we found that routine infection control failed to detect or prevent a community cluster involving 15 people who carried or were infected with ST22 MRSA. This was despite 2 fatal cases of bacteremia that were investigated using standard public health procedures [17] but were not linked to each other or the cluster until WGS was undertaken. Overall, epidemiological evidence was consistent with onward MRSA transmission in the community, although the precise circumstances under which this occurred could not be defined. Most patients were high users of primary care services including a GP leg ulcer clinic, although transmission through other unidentified contacts cannot be ruled out.

One case (P04) had a history of testing MRSA positive since December 2008, and WGS on available isolates confirmed carriage of the same MRSA lineage over a period of 34 months. The diversity within the isolates from P04 encompasses that of isolates from all other cases, potentially suggesting that persistent carriage in this case had contributed to spread of this lineage. Due to limited sampling, it is not possible to rule out reinfection, but the most recent common ancestor of these isolates would have dated to around 2006 (based on an SNP rate of ~3.5 SNPs/genome/year in ST22 [18]), consistent with carriage since that date. The important contribution of long-term MRSA carriers to transmission in hospitals has been shown previously [19], and is likely to be relevant in other settings. Decolonization of persistent carriers with chronic wounds such as leg ulcers is notoriously difficult, and rigorous infection control is required during treatment such as dressing changes when bacterial shedding can occur. MRSA was not isolated from staff or the environment at the GP practice during a point-prevalence survey, but this was performed a considerable period of time after the cluster had become established and was undertaken largely to identify modifiable factors.

MRSA ST22 is the most common MRSA lineage associated with healthcare-associated infection in the United Kingdom, and based on the higher overall prevalence of MRSA in hospitals vs the community in the last few decades, it has been assumed that the predominant directionality of spread is from hospitals into the community. Previous studies conducted in the United Kingdom have isolated ST22 from the community [20–22], but bacterial typing lacked sufficient resolution to infer transmission. To support this, *spa* genotyping of the cluster isolates in this study was undertaken, and based on the presence of a number of *spa* types it is unlikely that such typing would have identified this cluster. WGS has been used to confirm that transmission of a Panton-Valentine Leukocidin-positive, single locus variant of ST22 occurred from a special-care baby unit into the community, where it subsequently persisted [23, 24]. By contrast, the findings of our study suggest that most cases (9/13) associated with the MRSA cluster had either not attended a hospital or had at least 1 intervening sample that was MRSA negative in the 6 months prior to first MRSA detection. The majority of cases had links to clinic attendance in the community (in particular for ulcer care), providing genomic evidence for transmission of this typically nosocomial lineage within community rather than hospital settings.

A greater focus is needed to detect MRSA transmission in the community if overall MRSA bacteremia rates are to be further reduced. The role of infection prevention and control in the community will become increasingly relevant as initiatives are rolled out that increase delivery of care outside hospitals [25], and will require a review of the current predominantly hospital-centric structure of infection services [20]. Several methodological approaches could be considered. A low-cost passive surveillance option would count cases from submitting locations over time, associated with a defined threshold above which an investigation is triggered. However, the protracted period over which transmission occurred in the cluster described here meant rates of MRSA over time for the GP surgery were comparable with other similar practices. Consequently, the real-time analysis of epidemiological data from this practice is unlikely to have triggered an outbreak investigation. However, the addition of WGS allowed robust assessment of the relatedness of MRSA isolates and cases, and the implementation of surveillance WGS to control procedures may be a necessary tool if MRSA transmission is to be targeted by rapid interventions.

This study has a number of limitations. We cannot exclude that the outbreak may have been detected through other typing methods not undertaken here, such as pulsed-field gel electrophoresis. We did not undertake sampling for asymptomatic MRSA carriers in the wider community, which is likely to have underrepresented the extent of the cluster. Only a small proportion of the MRSA isolates from samples submitted by the GP surgery were available for sequencing, reducing the number of cases that could be included from the retrospective look-back. The study was not sufficiently powered to conduct a case-case design (cases with MRSA assigned to the cluster vs unrelated MRSA cases) to determine specific risk factors for MRSA acquisition, as comparison between practices was limited due to the variation in services provided. Finally, not all staff who may have been involved in the cluster were screened for MRSA due to staff turnover.

In conclusion, the detection of transmission and outbreaks associated with MRSA ST22 carriage and infection in the community is incomplete. In particular, this case study demonstrates the need to consider GP surgeries as transmission hotspots. Whereas WGS of all MRSA isolates from GP surgeries may not be cost-effective, this case study demonstrates how universal WGS of bacteremia isolates can detect relatedness and potential transmission events in settings that are not typically regarded as foci of transmission. Systematic WGS strategies could provide more accurate attribution of source, provide a mechanism for more efficient targeting of infection control, and lead to further reductions in the number of people who become colonized by, and go on to develop, MRSA bacteremia.

## Supplementary Data

Supplementary materials are available at *Clinical Infectious Diseases* online. Consisting of data provided by the authors to benefit the reader, the posted materials are not copyedited and are the sole responsibility of the authors, so questions or comments should be addressed to the corresponding author.

## Supplementary Material

Table_S1

Figure_S1

Figure_S2

## Notes

***Author contributions*.** M. S. T. and E. R. W. undertook the epidemiological and bioinformatic analyses with contributions from T. W., E. M. H., and F. C. Laboratory work was conducted by B. B. The public health investigation was managed by B. N. and N. B.; the investigation team also consisted of M. S. T., E. R. W., B. S., T. W., and S. J. P. Additional screening was undertaken on-site by B. S. and M. S. T; S. J. P. and J. P. supervised and managed the study. All authors had access to the data and read, contributed, and approved the final manuscript.

***Acknowledgments*.** We thank the practice manager and other staff at the GP surgery for their cooperation and involvement in this investigation, and Lynn Rodrigues and Rachel Thaxter for their contribution. We thank Jill Eastment, Dr M. Esteé Torok, Dr Sandra Reuter, and Dr Dorota Jamrozy for their advice and contributions. We acknowledge the Wellcome Trust Sanger Institute’s Core Sequencing Production Groups and Pathogen Informatics Group.

***Disclaimer.*** The study sponsors had no role in study design, data collection, data analysis, data interpretation, or writing of the report. The public health investigation was supported by Public Health England.

***Financial support*.** This work was supported by grants from the UK Clinical Research Collaboration Translational Infection Research Initiative and the Medical Research Council (grant number G1000803), with contributions to the grant from the Biotechnology and Biological Sciences Research Council, the National Institute for Health Research on behalf of the Department of Health, and the Chief Scientist Office of the Scottish Government Health Directorate (to S. J. P.); by a Healthcare Infection Society Major Research Grant (to S. J. P.); and by Wellcome Trust grant number 098051 awarded to the Wellcome Trust Sanger Institute. M. S. T. was a Wellcome Trust Clinical PhD Fellow. F. C. was supported by the Wellcome Trust (grant number 201344/Z/16/Z).

***Potential conflicts of interest.*** N. M. B. is on the advisory board for Discuva Ltd. All other authors report no potential conflicts. All authors have submitted the ICMJE Form for Disclosure of Potential Conflicts of Interest. Conflicts that the editors consider relevant to the content of the manuscript have been disclosed.
