# Supplementary material for: Investigation of a Cluster of Sequence Type 22 Methicillin-Resistant Staphylococcus aureus Transmission in a Community Setting
Source: Clin Infect Dis. 2017 Oct 25;65(12):2069–77. doi: 10.1093/cid/cix539 (PMC5850418; doi:10.1093/cid/cix539)

Figure S1. Phylogenetic analysis of 29 MRSA ST22 isolates from 15 cases linked to a GP surgery. Midpoint rooted maximum likelihood tree based on SNPs in the core genome. Colored bars indicate *spa* genotype: red, t294; blue, t032; yellow, t379; orange, t1302; purple t492; grey, not typable/not done.

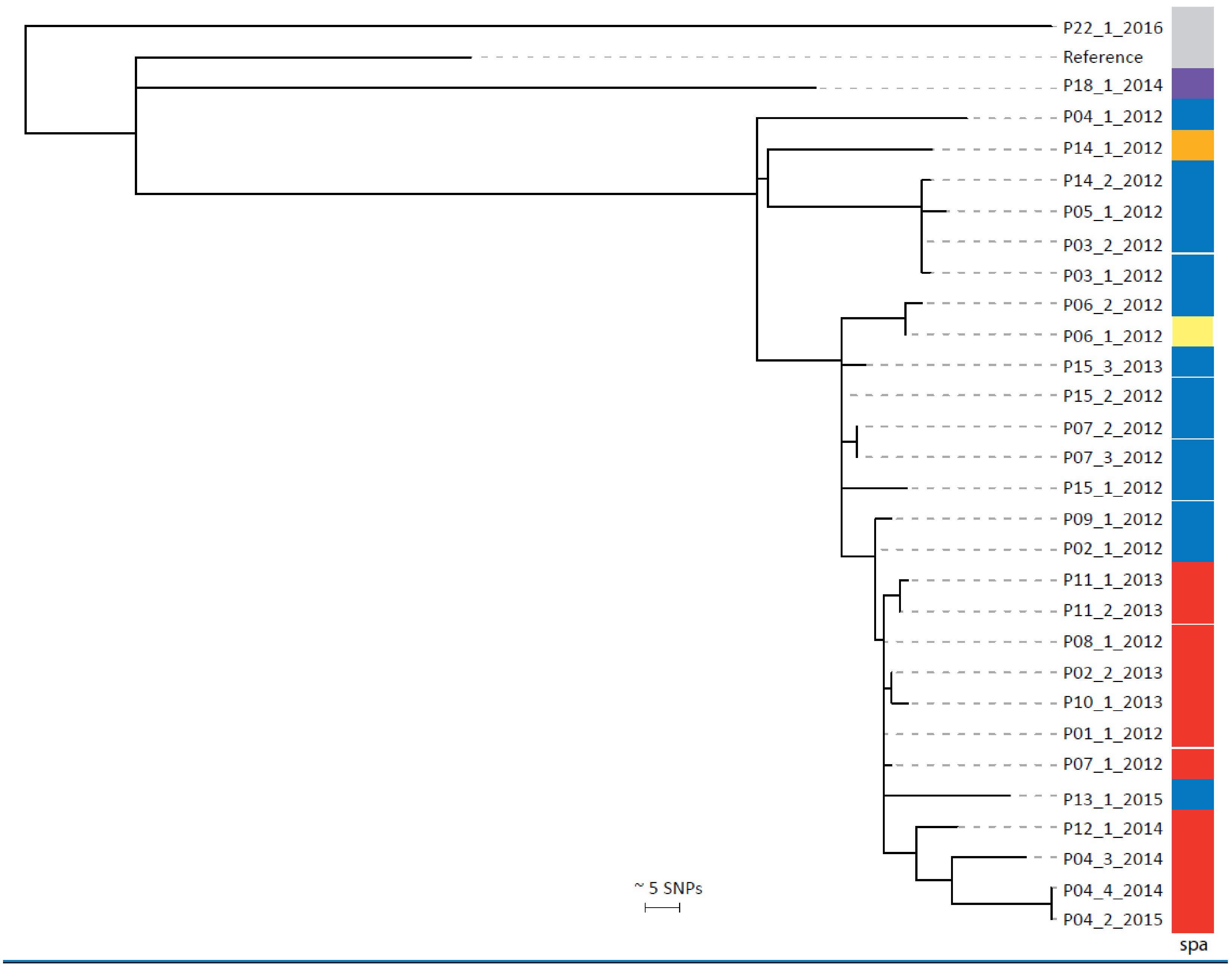

Supplement: Figure_S1 [file cix539_suppl_figure_s1.pdf]
