# Supplementary material for: Investigation of a Cluster of Sequence Type 22 Methicillin-Resistant Staphylococcus aureus Transmission in a Community Setting
Source: Clin Infect Dis. 2017 Oct 25;65(12):2069–77. doi: 10.1093/cid/cix539 (PMC5850418; doi:10.1093/cid/cix539)

Figure S2. Comparative incidence rate of MRSA for the study GP surgery and four comparable general practices. Bold line represents practice studied. Practices not labelled to maintain organisational anonymity.

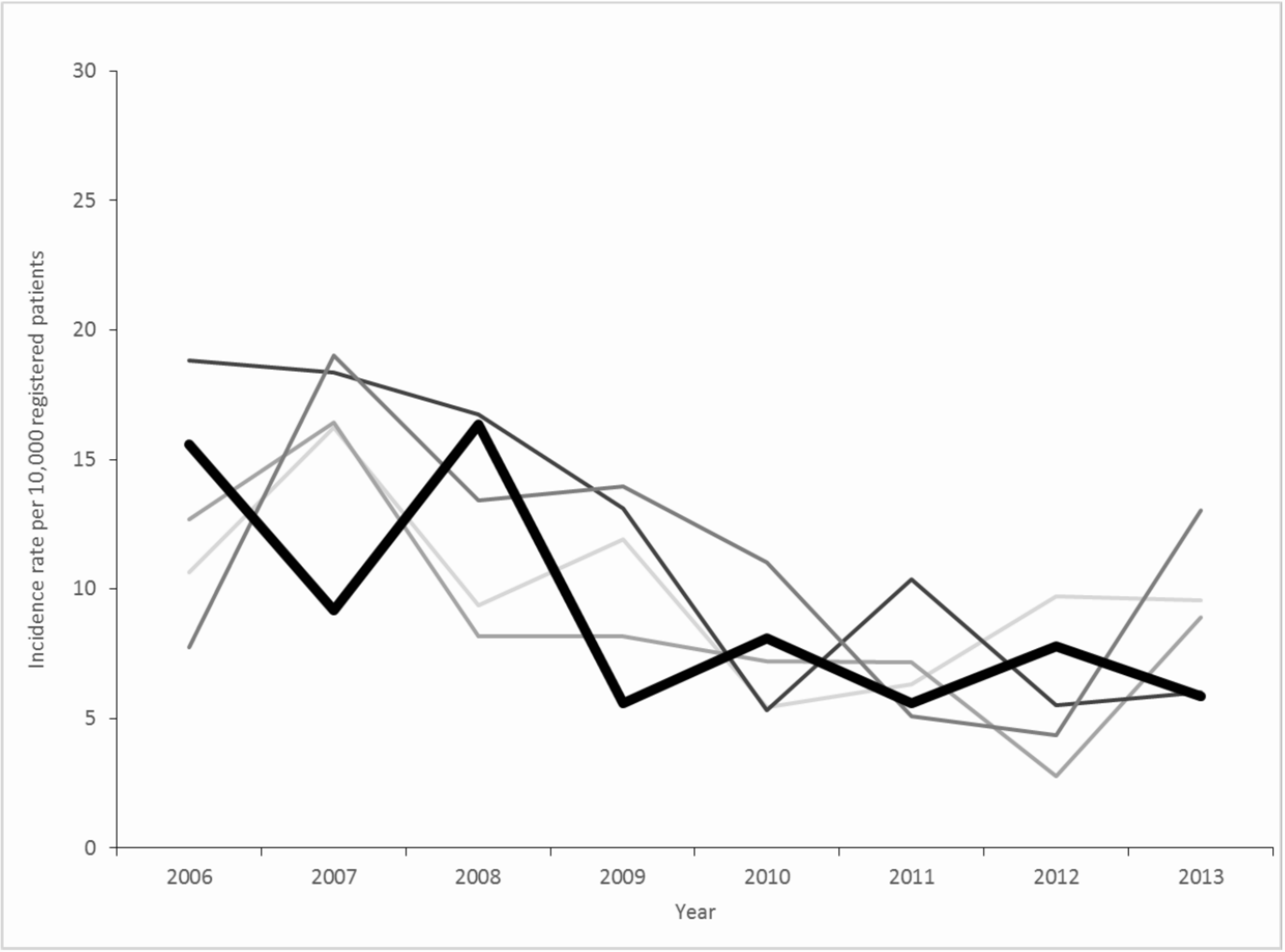

Supplement: Figure_S2 [file cix539_suppl_figure_s2.pdf]
